# The quality of acute intensive care and the incidence of critical events have an impact on health-related quality of life in survivors of the acute respiratory distress syndrome – a nationwide prospective multicenter observational study

**DOI:** 10.3205/000277

**Published:** 2020-01-20

**Authors:** Thomas Bein, Steffen Weber-Carstens, Christian Apfelbacher, Susanne Brandstetter, Sebastian Blecha, Frank Dodoo-Schittko, Magdalena Brandl, Michael Quintel, Stefan Kluge, Christian Putensen, Sven Bercker, Björn Ellger, Thomas Kirschning, Christian Arndt, Patrick Meybohm, Florian Zeman, Christian Karagiannidis

**Affiliations:** 1 Department of Anaesthesia & Operative Intensive Care, University Hospital Regensburg, Germany; 2 Department of Anaesthesiology and Intensive Care Medicine, Charité – University Medicine Berlin, Germany; 3 Institute for Social Medicine and Health Economy, Magdeburg University, Magdeburg, Germany; 4 Medical Sociology, Institute of Epidemiology and Preventive Medicine, University of Regensburg, Germany; 5 Department of Anaesthesiology, Emergency and Intensive Care Medicine, University Medicine, Göttingen, Germany; 6 Department of Intensive Care Medicine, University Medical Centre, Hamburg-Eppendorf, Germany; 7 Department of Anaesthesiology and Operative Intensive Care, University Hospital Bonn, Germany; 8 Department of Anaesthesiology and Intensive Care Medicine, University Hospital Leipzig, Germany; 9 Department of Anaesthesiology and Intensive Care, Klinikum Dortmund, Germany; 10 Department of Anaesthesiology and Intensive Care, University Hospital Mannheim, Germany; 11 Department of Anaesthesiology and Operative Intensive Care, University Hospital Marburg, Germany; 12 Department of Anaesthesiology, Intensive Care Medicine, and Pain Therapy, University Hospital Frankfurt, Germany; 13 Center of Clinical Studies, Regensburg University Medical Center, Regensburg, Germany; 14 Department of Pneumology and Critical Care Medicine, Cologne-Merheim Hospital, ARDS and ECMO Centre, Kliniken der Stadt Köln, Witten/Herdecke University Hospital, Cologne, Germany

**Keywords:** acute respiratory distress syndrome, quality of care, critical events, health-related quality of life, return to work

## Abstract

**Background:** Initial treatment (ventilator settings, rescue therapy, supportive measures), and prevention of critical events improve survival in ARDS patients, but little data exists on its effect on health-related quality of life (HRQOL) and return to work (RtW) in survivors. We analyzed the association of the intensity of treatment at ARDS onset and the incidence of critical events on HRQOL and RtW a year after ICU discharge.

**Methods:** In a prospective multi-centre cohort study, the intensity of treatment and the incidence of critical events were determined at 61 ICUs in Germany. At 3, 6, and 12 months, 396 survivors reported their HRQOL (Short-Form 12) and RtW. The parameters of the intensity of acute management (lung protective ventilation, prone position, hemodynamic stabilization, neuromuscular blocking agents), and critical events (hypoxemia, hypoglycemia, hypotension) were associated with HRQOL and RtW.

**Results:** Patients ventilated at ARDS onset with a low tidal volume (VT≤7 ml/kg) had higher arterial carbon dioxide levels (PaCO_2_=57.5±17 mmHg) compared to patients ventilated with VT>7ml/kg (45.7±12, p=0.001). In a multivariate adjusted dichotomized analysis, a better mental 3-month SF-12 was observed in the higher VT-group (mean 43.1±12) compared to the lower VT-group (39.5±9, p=0.042), while a dichotomized analysis for driving pressures (≤14 mbar vs >14 mbar) did not show any difference neither in PaCO_2_ levels nor in HRQOL parameters. A decrease in the mental (6-month: 40.0±11 vs 44.8±13, p=0.038) and physical SF-12 (12-month: 38.3±11 vs 43.0±13, p=0.015) was reported from patients with hypoglycemia (blood glucose <70 mg/dl) compared to those without hypoglycemic episodes. More frequent vasopressor use with mean arterial pressure ≥65 mmHg was associated with an impaired physical SF-12 (6-month: 38.8±10) compared to less vasopressor use (43.0±11, p=0.019).

**Conclusions:** In acute management of ARDS, a lower VT strategy associated with hypercapnia, as well as the frequent usage of catecholamines and the management of blood glucose may influence short-term HRQOL of survivors. The awareness of these findings is of clinical importance for the acute and post-ICU care.

## Zusammenfassung

**Hintergrund:** Die Qualität der aktuellen Behandlungsstrategie (Beatmungseinstellung, rescue-Maßnahmen, unterstützende Intensivtherapie) sowie die Vermeidung kritischer Ereignisse verbessern das Überleben von Patienten mit schwerem akuten Lungenversagen (ARDS). Es ist bisher wenig bekannt darüber, ob eine solche Behandlungsqualität auch einen Einfluss auf die gesundheitsbezogene Lebensqualität (health-related quality of life, HRQOL) und die Rückkehr in das Arbeitsleben (return to work, RtW) der Überlebenden hat. Wir untersuchten eine mögliche Beziehung zwischen akuter Behandlungsqualität, HRQOL und RtW ein Jahr nach Überleben eines ARDS.

**Methoden:** Die prospektiv-multizentrische Studie wurde in 61 Intensivstationen (ICUs) in Deutschland durchgeführt. Drei, sechs und zwölf Monate nach Verlegung von der ICU gaben 396 Überlebende über ihre HRQOL (Short-Form-12) und RtW Auskunft. Die Parameter der akuten Behandlungsqualität (lungenprotektive Beatmungseinstellung, Gradient zwischen endexpiratorischem und inspiratorischem Beatmungsdruck (driving pressure), Einsatz von Bauchlagerung und/oder Muskelrelaxantien, hämodynamische Stabilisierung) und die Häufigkeit kritischer Ereignisse (Hypoxämie, Hypoglykämie, Hypotension) wurden mit HRQOL und RtW assoziiert.

**Ergebnisse:** Patienten, die zu Beginn des ARDS mit einem niedrigen Tidalvolumen (VT≤7 ml/kg) beatmet wurden, wiesen höhere arterielle Karbondioxid-Werte auf (PaCO_2_=57.5±17 mmHg) im Vergleich zu Patienten mit höherem VT>7 ml/kg (PaCO2 45.7±12, p=0.001). In einer multivariaten adjustierten dichotomisierten Analyse wurde eine bessere 3-Monats-Lebensqualität in der mentalen Domäne des SF-12 in der Gruppe mit einem höheren VT gefunden (Mittelwert 43.1±12) im Vergleich zu den mit niedrigerem VT beatmeten Patienten (39.5±9, p=0.042); für eine dichotomisierte Analyse des driving pressure (≤14 mbar vs >14 mbar) wurde weder für PaCO_2_ noch für HRQOL ein Unterschied gefunden. Eine Einschränkung der mentalen (6 Monate: 40.0±11 vs 44.8±13, p=0.038) und körperlichen SF-12 Domäne (12 Monate: 38.3±11 vs 43.0±13, p=0.015) wurde für Patienten berichtet, die während der akuten Behandlung eine Hypoglykämie (Blutglukose-Wert <70 mg/dl) aufgewiesen hatten im Vergleich zu Patienten ohne dokumentierte Hypoglykämie-Episode. Ein erhöhter Einsatz von Vasopressoren zur Stabilisierung des arteriellen Blutdruckes (Ziel: mittlerer Wert ≥65 mmHg) war mit einer Einschränkung der körperlichen Domäne des SF-12 verknüpft (6 Monate: 38.8±10) im Vergleich zu weniger Vasopressoren-Einsatz (43.0±11, p=0.019).

**Schlussfolgerung:** Im Rahmen des akuten Managements von ARDS-Patienten führten sowohl die empfohlene lungenprotektive Beatmungsstrategie mit niedrigem Tidalvolumen (<7 ml/kg) und Hyperkapnie, als auch der Einsatz von Vasopressoren zur Blutdruckstabilisierung zu einer größeren Einschränkung der Lebensqualität im ersten Jahr nach Überleben. Die Vermeidung kritischer Ereignisse (Hypoglykämie) war mit besserer Lebensqualität verknüpft. Diese Ergebnisse sind für die Nachsorge-Strategie von Patienten nach ARDS von Bedeutung.

## Background

Over the past 25 years, patients suffering from ARDS have witnessed a decrease in mortality rates due to improved ventilatory support and other strategies. Although evidence for certain treatment strategies in the ARDS management is based on high-quality scientific results, translating the research into routine has been challenging [1]. Most importantly, low tidal volume ventilation [2], prone position [3], or the application of neuromuscular blocking agents in the early phase [4] were found to improve survival. Furthermore, the occurrence of critical events (hypoxemia [5], hypoglycemia [6], and hypotension [7]) during the early ‘vulnerable’ phase is associated with increased mortality. Thus, the adherence to evidence-based acute care quality and the prevention of critical events are accepted markers for value in intensive care that promote a successful treatment with improved survival.

In recent years – in addition to mortality – a growing interest in the long-term outcomes of ARDS patients with a focus on health-related quality of life (HRQOL), functional autonomy, satisfaction, everyday competence and return to work (RtW) [8], [9], [10] has been observed. Various studies conducted with ARDS survivors have revealed a substantial incidence of mental disorders [11], [12], [13], reduced social integration or RtW [14]). In the study by Pfoh et al. [15], around 69% of ARDS survivors experienced physical decline, but the severity of disease was not strongly associated with the severity of decline. These results were summarized in a recent review [16].

Since the quality of acute management in clinical practice and the prevention of critical events are associated with improved survival, we were interested in the effects of applying these evidence-based quality markers on the one-year outcome in patients surviving ARDS. The hypothesis underlying our current study is that in ARDS patients subsequently treated with the recommended therapeutic strategies and in those prevented from critical events, a better HRQOL and a higher rate of RtW will be observed.

## Methods

### Study design and sample

This study is a partial analysis of the DACAPO study, which is a prospective multi-center cohort study. The study protocol and cohort profile have been described in detail elsewhere [17], [18]. In short, ARDS patients were recruited from 61 intensive care units (ICUs) all over Germany between 2014 and 2017. The inclusion criteria were that the patients were adults, had an ARDS diagnosis according to the ‘Berlin’ definition [19] and provided written informed consent (either by themselves or their caregivers/legal guardians), no exclusion criteria were given. The participating centres were urged to manage the patients in accordance with the actually proposed best evidence regarding ARDS management (lung protective ventilation, other supportive measures), hemodynamic support and prevention or management of critical events. During the ICU stay, characteristics relating to medical aspects and care were recorded by trained staff using electronic case report forms. The patients were contacted by mail after 3, 6, and 12 months of discharge from the ICU and requested to complete self-report questionnaires. The study was approved by the Ethics Committee of the University of Regensburg (file number: 13-101-0262) as well as by the ethics committees responsible for the participating hospitals.

### Measurements

#### Outcomes

The HRQOL and RtW outcomes were assessed at each follow-up by the 12-item Short Form self-reported questionnaire (SF-12) [20]. The published scoring algorithm results in the Physical Component Summary (PCS-12) and Mental Component Summary (MCS-12) scores. The scores range from 0–100 (with 50 being the mean value for the general German population [21]). The RtW was only assessed in persons who had been working prior to their ICU admission. ARDS survivors with valid responses up to 13 months after discharge from the ICU were enrolled in the analysis. Mortality was not included as an outcome parameter for qualitative or adjusted analyses since our study did not fulfill the criteria for a ‘registry-study’, and some patients with poor prognosis were not enrolled by some centres.

#### Exposures

The patterns of acute management were retrospectively assessed and analysed as follows: Lung protective ventilation was characterized by the *tidal volume* (VT=ml/kg predicted body weight, PBW) at the onset of ARDS (first reported value when diagnosis was made), and it was dichotomized for analysis =VT≤7 ml versus VT>7 ml, since in a large international multicenter observational study (LUNG SAFE [22]), a VT≈7 ml/kg was the lung-protective level of choice in most study patients. Additionally, the *driving pressure* (ΔP=the difference between plateau pressure and total positive endexpiratory pressure, PEEP) was simultaneously recorded and it was dichotomized =ΔP≤14 mbar versus >14 mbar, because a strong association between ΔP≤14 mbar and improved survival was found [23]. The *prone position* and the application of *neuromuscular blocking agents* for more than 3 hours were recorded and dichotomized in ‘yes’ versus ‘no’ for each individual patient. The occurrence of critical events in the recruited ARDS patients was noted by the respective study centre: Hypoxemic episodes (SaO_2_<85% for at least 5 minutes), hypoglycemia (blood glucose <70 mg/dl), and the simultaneous recording of mean arterial pressure (MAP), while targeting a level of MAP=65 mmHg. This variable was dichotomized for analysis versus MAP<65 mmHg, and the ICUs used vasopressor therapy (norepinephrine) to avoid ‘hypotension’ (MAP<65 mmHg).

#### Potentially confounding variables

The variables relating to age, sex, severity of disease (SAPS II score), and body mass index (BMI) were assessed as potentially confounding variables. Comorbidities (except pre-existing psychiatric disorders) and the severity of chronic diseases were not recorded specifically, since the most prognostic relevant illnesses and the types of ICU admission are part of the SAPS II score. The general medical characteristics were captured with regard to Sequential Organ Failure Assessment (SOFA) [24] score and BMI (kg/m²). Approximately 30% of the ARDS patients had received extracorporeal membrane oxygenation (ECMO), and therefore we did not include the PaO_2_/FIO_2_ ratio for multivariable adjusted analysis, since this ratio may not have been an adequate surrogate parameter for the severity of ARDS under ECMO.

### Statistical analysis

Patient characteristics are presented as median (interquartile range, IQR) for continuous data, and as absolute and relative frequencies for categorical data. The impact of VT, ΔP, prone position, neuromuscular blocking agents, hypoxemia, hypoglycemia, and MAP on the two dimensions of HRQOL was analyzed by using an analysis of covariance (ANCOVA). Each variable was analyzed in a separate model, all adjusted for SOFA (without Glasgow Coma Scale), body mass index, age and sex. Estimated marginal means with corresponding 95% confidence intervals are presented as effect estimates. The impact of the listed exposures on 12-month RtW were analyzed by means of logistic regression models, also adjusted for SOFA (without Glasgow Coma Scale), BMI, age and sex. Odds ratios (OR) and 95% confidence intervals are presented as effect estimates. In this prospective multi-centre study, some cases of missing data at random occurred. In these cases, a partwise/pairwise deletion of variables was performed. A p-value of <0.05 was considered statistically significant for all analyses. No adjustments for multiple comparisons were made due to the exploratory nature of these additional analyses of the DACAPO study. Analyses were performed using SPSS 25.0 (BM, New York, NY, USA).

## Results

**Figure 1:**
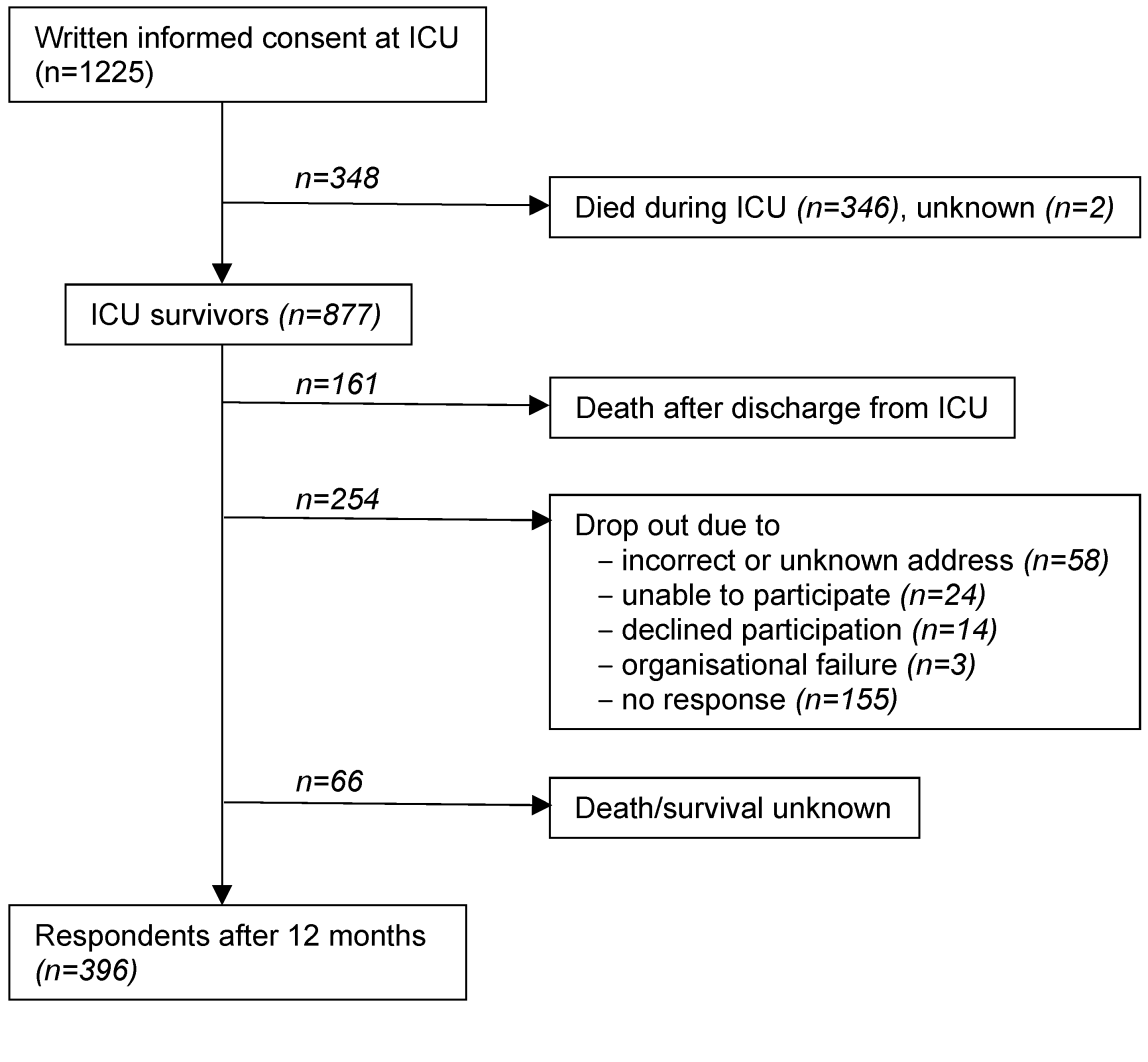
Patient flow. For all follow-up patients, survival/death was assessed via local municipial population registries.

Written informed consent was obtained for 1,225 ARDS patients from 61 ICUs, of these 28.2% (346 patients) died in the ICU, thus the post-ICU survivor DACAPO cohort comprised 877 patients, of which 481 patients (54.8%) could not be followed (death, incorrect address, inability to participate, unknown reasons (Figure 1 (Fig. 1))). 396 patients were successfully followed up to 12 months.

The general characteristics of the respondents in the 12-month follow-up are described in Table 1 (Tab. 1). The median age at ICU admission was 56 years, two-thirds of the 12-month respondents were male, and the median length of ICU stay was 23 days. The majority of patients suffered from moderate or severe ARDS. A little over half of the patients working prior of the study returned to work after one year.

The characteristics of the acute management in the 12-month survivors are presented in Table 2 (Tab. 2). A tidal volume ≤7 ml/kg at the time of diagnosis of ARDS and a ‘lung protective’ ΔP≤14 mbar were applied in nearly half of the patients (Table 2 (Tab. 2), a). 47% of the patients were ventilated in prone position and 23% had received neuromuscular blocking agents during acute treatment. Regarding critical events, 27% of the patients suffered from hypoxemia, while the prevalence of hypoglycemia or hypotension was approximately 15% (Table 2 (Tab. 2), b).

### Quality of acute management and health-related quality of life

**Figure 2:**
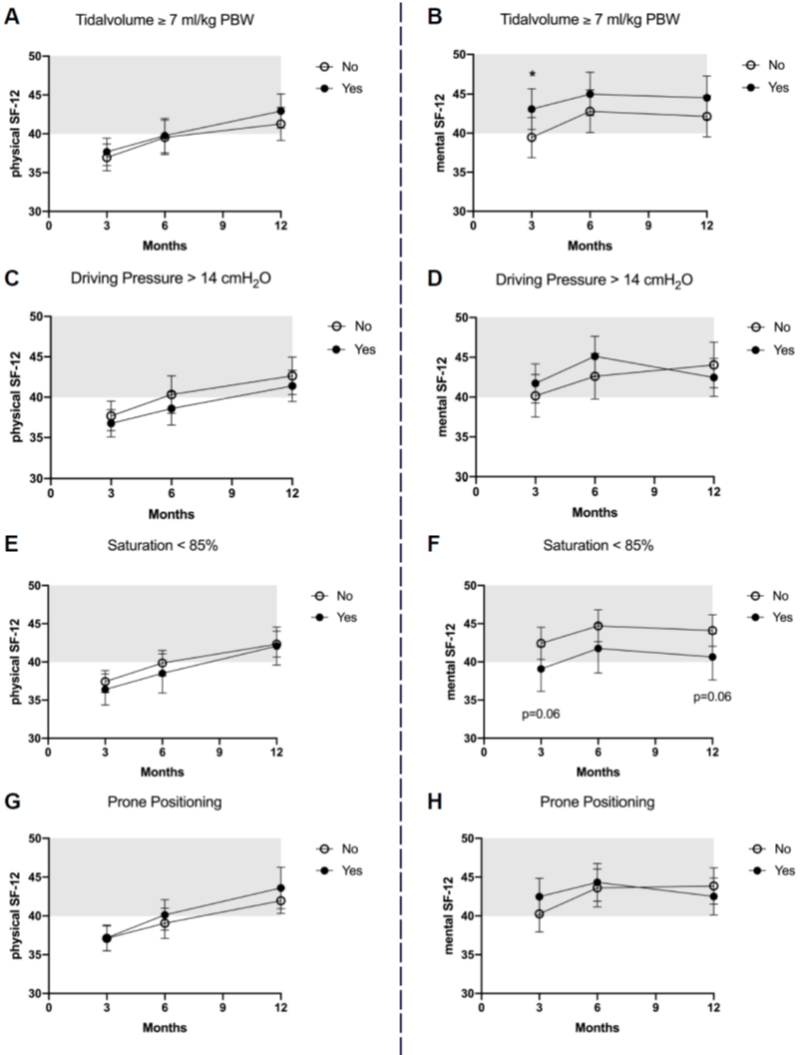
HRQOL in ARDS survivors after 3, 6 and 12 months for the physical and mental component of the SF-12 score in regard to tidal volume ≥7 ml/kg pBw (A and B), driving pressure <14 cm H_2_O (C and D), desaturation <85% for more than 5 minutes (E and F) and prone positioning (G and H). The mean score of the population is 50, whereas one standard deviation is marked in grey.

**Figure 3:**
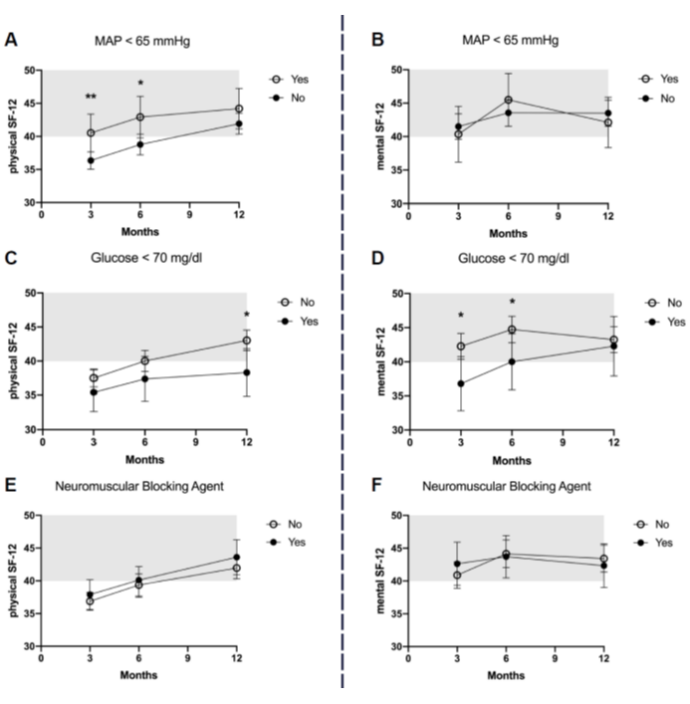
HRQOL in ARDS survivors after 3, 6 and 12 months for the physical and mental component of the SF-12 score in regard to mean arterial pressure <65 mmHg (A and B), glucose <70 mg/dl (C and D), neuromuscular blocking agent for more than 3 hours (E and F). The mean score of the population is 50, whereas one standard deviation is marked in grey.

The 12-month ARDS survivors reported a mean physical SF-12=42±11 and a mental SF-12=44±13. The association between acute management or critical events and HRQOL is presented in Figure 2 (Fig. 2) and Figure 3 (Fig. 3), and the results of the multiple adjusted analysis are demonstrated in Table 3 (Tab. 3). Ventilation with a VT>7ml/kg was associated with lower PaCO_2_ levels, and up to 3 months these patients reported a significantly better mental SF-12. No differences in HRQOL were found for ΔP, prone position or neuromuscular blocking agents. The patients with reported hypoxemia during acute care showed a trend towards impaired mental health status up to 12 months after ICU treatment compared to patients without any hypoxemic episode. The occurrence of ‘hypotensive’ episodes with less catecholamine use was associated with a significantly better physical SF-12 up to six months after release from the ICU, hypoglycemic episodes were associated with a marked decrease in the mental SF-12 and an impaired physical function up to 12 months.

### Correlation of quality of acute management with return to work

The effects of acute management and critical events on RtW (unadjusted model and multiple analysis) are presented in Table 4 (Tab. 4). No associations between parameters of acute care or between critical events and the possibility of returning to work were found in the adjusted model.

## Discussion

In this study, we determined the influence of initial treatment and critical events on the one-year quality of life and return to work in ARDS survivors, since it has been shown that these parameters have an impact on mortality, but in terms of the *quality of survival* no data exists. The main results are the following:

1. Patients ventilated with a VT>7ml/kg compared to ≤7 ml had significantly lower PaCO_2_ levels, and they reported a significant better short-term mental SF-12 after 3 months.
2. The prevention of hypotensive episodes by vasopressor use (MAP≥65 mmHg) was associated with a significantly impaired physical SF-12 up to six months.
3. Hypoglycemia was associated with a marked decrease in the mental (6-month) and physical (12-month) SF-12.
4. Patients with reported hypoxemic incidents showed a trend towards an impaired mental health status up to 12 months after ICU discharge compared to patients without hypoxemic episodes.

### Quality of acute ARDS management and association with health-related outcome

In our study, the low VT ventilation was associated with a 3-month impairment of the mental SF-12 compared to patients ventilated with a higher VT. The interpretation of this finding – since it is accepted that low VT will improve survival – might be complex. Low VT strategy is often combined with permissive hypercapnia, but ‘safe’ levels of hypercapnia and acidemia are still unknown [25]. In a secondary analysis of 1899 ARDS patients from three prospective cohort studies performed in 927 ICUs [26], patients with hypercapnia (PaCO_2_≥50 mmHg) had higher complication rates, more organ failures, and worse outcomes, and severe hypercapnia was independently associated with higher ICU mortality. In line with previous results, our study demonstrates a marked increase in PaCO_2_ in patients ventilated with less than 7 ml/kg. However, avoidance of hypercapnia on the expense of non-protective ventilation was associated with short-term improved HRQOL. While some protective effects of acute hypercapnic acidosis in experimental studies are expressed, also several deleterious consequences (pulmonary hypertension, infection, inflammation, diaphragm dysfunction, and cerebral ischemia) are addressed. It is still a matter of controversial debate whether and to what extent acute hypercapnic acidosis might impair several physiologic functions on a macro- and microvascular level [27]. During acute hypercapnia, a marked dysregulation of cerebral autoregulation exists [28], which is a mechanism that maintains cerebral blood flow stable despite fluctuating perfusion pressures. An adjusted analysis of a cardiac arrest registry [29] demonstrated that hypercapnia (PaCO_2_≥50 mmHg during the first 24 h after arrest) was an independent predictor of poor neurological function at hospital discharge (OR 2.20 [95% CI 1.03–4.71). Based on these findings and our results, further investigations should focus on the role of hypercapnia – although life-saving in ARDS – on HRQOL in ARDS survivors. In our study, interestingly a ‘lung protective’ driving pressure had no impact on acute PaCO_2_ levels and mental HRQOL. This additional result may underline our assumption that more the hypercapnia than the tidal volume itself is responsible for the observed effects.

### The association of critical events with health-related outcome

The prevention of critical events is an important goal for quality improvement. Mikkelsen et al. [6] assessed the long-term neuropsychological function of ARDS survivors in relation to parameters of acute care. They found that a lower arterial oxygenation (SaO_2_<92%) was associated with cognitive and psychiatric impairment. In our study – consistent with these findings – ARDS survivors with hypoxemia reported a sustained trend towards an impaired mental HRQOL at 3 and 12 months after ICU compared to patients without any hypoxemic episode. This should draw our attention to avoid desaturation in the initial and vulnerable phase of ARDS. Furthermore, hypoglycemia undergoing acute treatment is associated with increased mortality [7]. Interestingly, our study could demonstrate that hypoglycemia in surviving patients also decreases mental (6-month) and physical (12-month) HRQOL. In consequence, patients surviving a hypoglycemic or hypoxemic episode should undergo an advanced specific neurologic evaluation and/or rehabilitation early after transferal from the ICU.

Another interesting finding from our study is that in ARDS survivors, the correction of MAP during acute care above 65 mmHg by vasopressors was followed by an impaired 3-month physical HRQOL. Although difficult to determine, according to our results we hypothesize that the usage of catecholamines was responsible for the observed effect. A recent randomized study in patients with septic shock [30] and a pooled analysis [31] on pressure targets for vasopressor therapy found that targeting higher blood pressure may increase mortality in patients, and lower blood pressure targets were not associated with adverse events; an evident MAP target is still controversially discussed [32]. Furthermore, several negative non-hemodynamic effects of catecholamines (immunosuppression, alteration of glucose metabolism and/or decrease splanchnic perfusion, and gastrointestinal motility) were identified as a consequence of the ubiquitous presence of their receptors [33]. In a retrospective case-cohort study of critical care transport patients with systolic blood pressure <70 mmHg [34], bolus doses of epinephrine increased blood pressure, but the cohort treated with these boluses had a higher mortality than the cohort without boluses. So far, no data exists in terms of the effects of inotropes on the physical and mental status in survivors of critical illness. Based on our data, we take into consideration that a strategy of vasopressor infusion aimed at higher MAP targets might influence HRQOL, though our study could not assess the cumulative dose of vasopressors in each patient.

Furthermore, a second critical event, i.e. hypoglycemia (blood glucose <70 mg/dl) was associated with a marked decrease in the mental and physical health status, and this finding is in accordance with a recent case-control study on 53 pediatric ICUs [35] in critically ill children, where any hypoglycemia (blood glucose levels <60 mg/dl) was associated with worse short-term outcomes. Furthermore, in a prospective cohort study in 104 consecutive survivors of acute lung injury by Dowdy et al. [36], hypoglycemia in the ICU was associated with an increased risk of positive screening for depression during early recovery from acute lung injury (ALI).

The strengths of the present study include the prospective design with three follow-ups, the detailed collection of data on both acute management and critical events, and a large data base of included patients with ARDS from 61 hospitals all over Germany. On the other hand, a considerable proportion of patients were lost to follow-up. Loss to follow-up is a permanent problem in observational studies investigating long-term survivors [37], and it may compromise the external validity of the findings or introduce attrition bias. Furthermore, a certain proportion of missing data was managed by partial or pairwise exclusion. Another potential biasing factor is the fact that we reported the data of post-ICU health care use (stationary, ambulatory, and rehabilitation [38]), which might have influenced HRQOL, but we did not integrate these data as potentially confounding parameters into the multivariable analysis.

## Conclusion

The current study demonstrates that patterns of treatment at the onset of ARDS and the prevention of critical events influence long-term outcome of the survivors. In particular, hypoxemic episodes, and hypoglycemia, are associated with reduced HRQOL. Hypercapnia associated with low tidal volume ventilation (≤7ml/kg) was related temporarily to the mental health status, which should be determined in detail in upcoming studies. Interestingly, a hemodynamic management to maintain MAP≥65 mmHg using vasopressors was associated with impaired HRQOL. The importance of these findings in light of lung protection and hemodynamic stabilization has to be determined.

## Notes

### Ethics approval and consent to participate

The study was approved by the Ethics Committee of the University of Regensburg (file number: 13-101-0262) as well as by the ethics committees responsible for the participating hospitals.

### Availability of data and material

All data collected for the study (de-identified participant data, data dictionary, or other specified data set) will be made available to others upon request.

### Competing interests

This study is part of the DACAPO study which was funded by a research grant from the German Federal Ministry of Education and Research (01GY1340). Grant holders were TB (University Hospital Regensburg, principal investigator) and CA (University of Regensburg, co-principal investigator). SuB, FDS, MB and SeB were funded by this grant for parts of or the entire study period. All other authors received payments from the grant to support patient recruitment. 

TB, CK, MQ, SK, CP, SvB, BE, TK, CAr, PM, and SWC are members of the German ARDS Network. TB: received honoraria for lectures from Xenios, Germany. MQ: received honoraria for lectures from Maquet and Xenios, Germany. All other authors declare: no relationships/conditions/circumstances that present a potential conflict of interest.

### Funding

German Federal Ministry of Education and Research (01GY1340)

### Authors’ contributions

TB, CAp, SuB, SeB, FDS, SWC, and MQ conceived the study, FDS, MB, and SuB were responsible for methods and statistics. Additionally, CK and SWC contributed to the writing team. SK, CP, SvB, BE, TK, CAr, PM were involved in recruitment and practical implementation of the study. All authors reviewed and edited the article.

### Trial registration

Clinicaltrials.gov: NCT02637011

### Acknowledgement

See Attachment 1

## Supplementary Material

Acknowledgement

## Figures and Tables

**Table 1:**
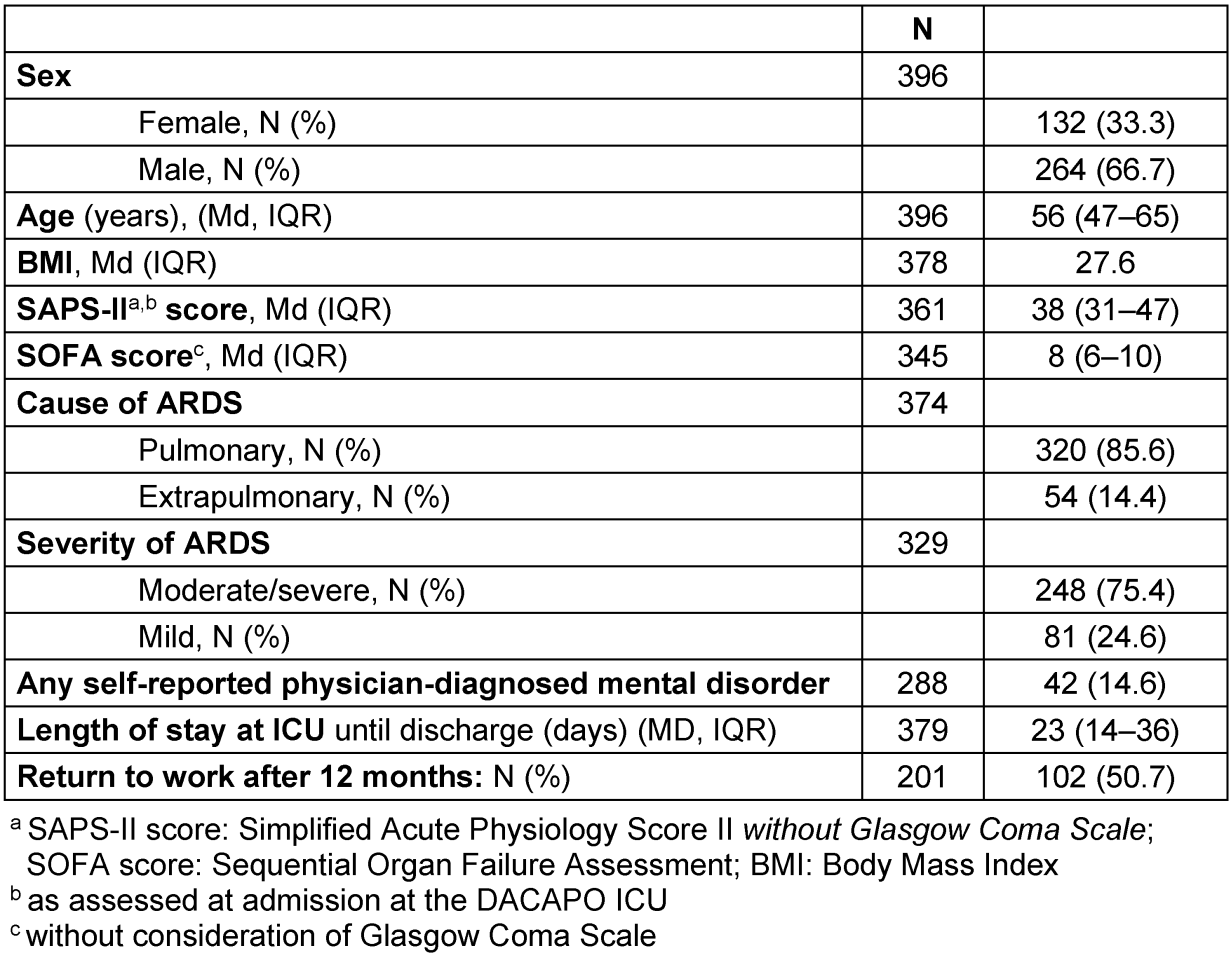
General characteristics of 12-month respondents

**Table 2:**
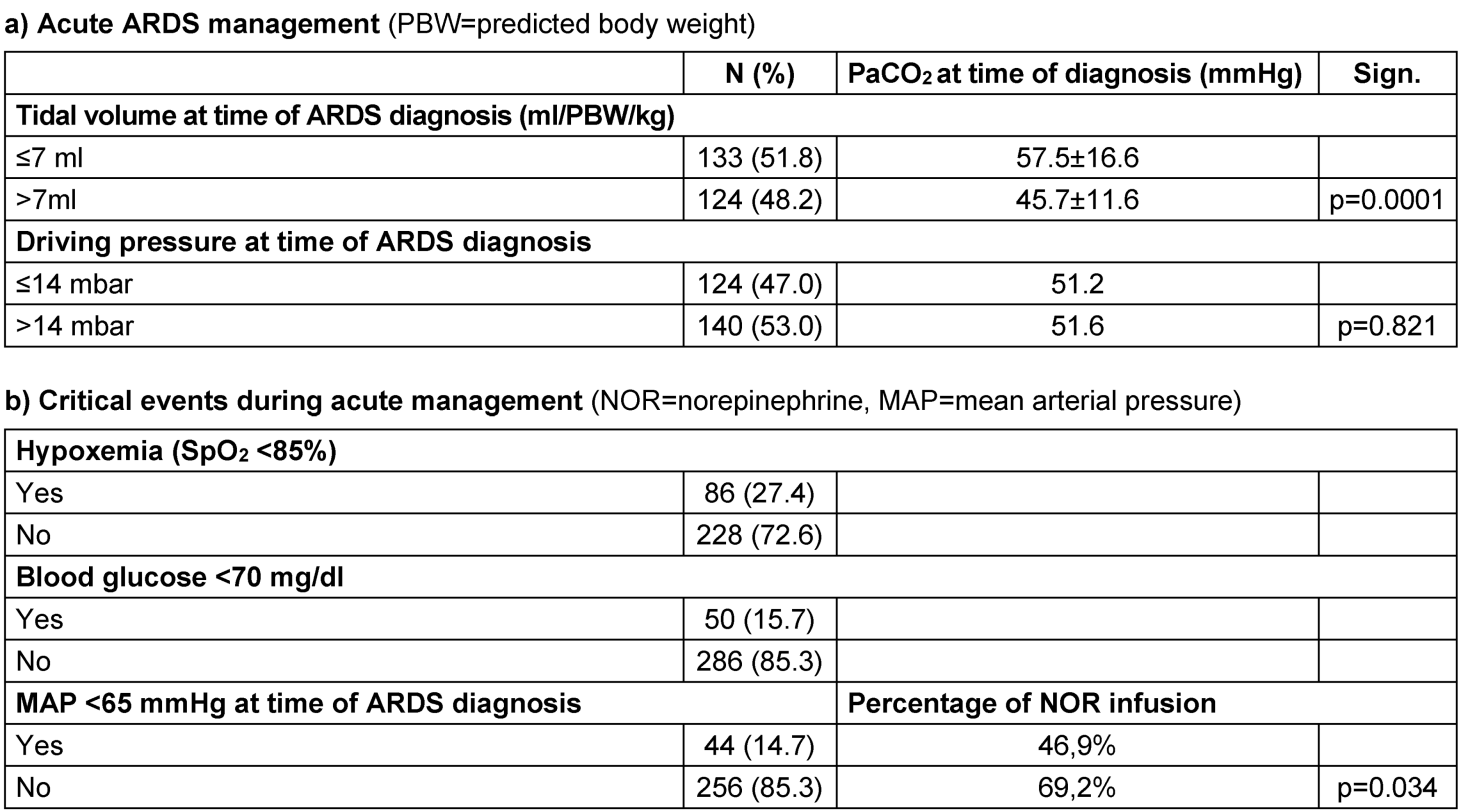
Characteristics of acute management of the 12-month respondents

**Table 3:**
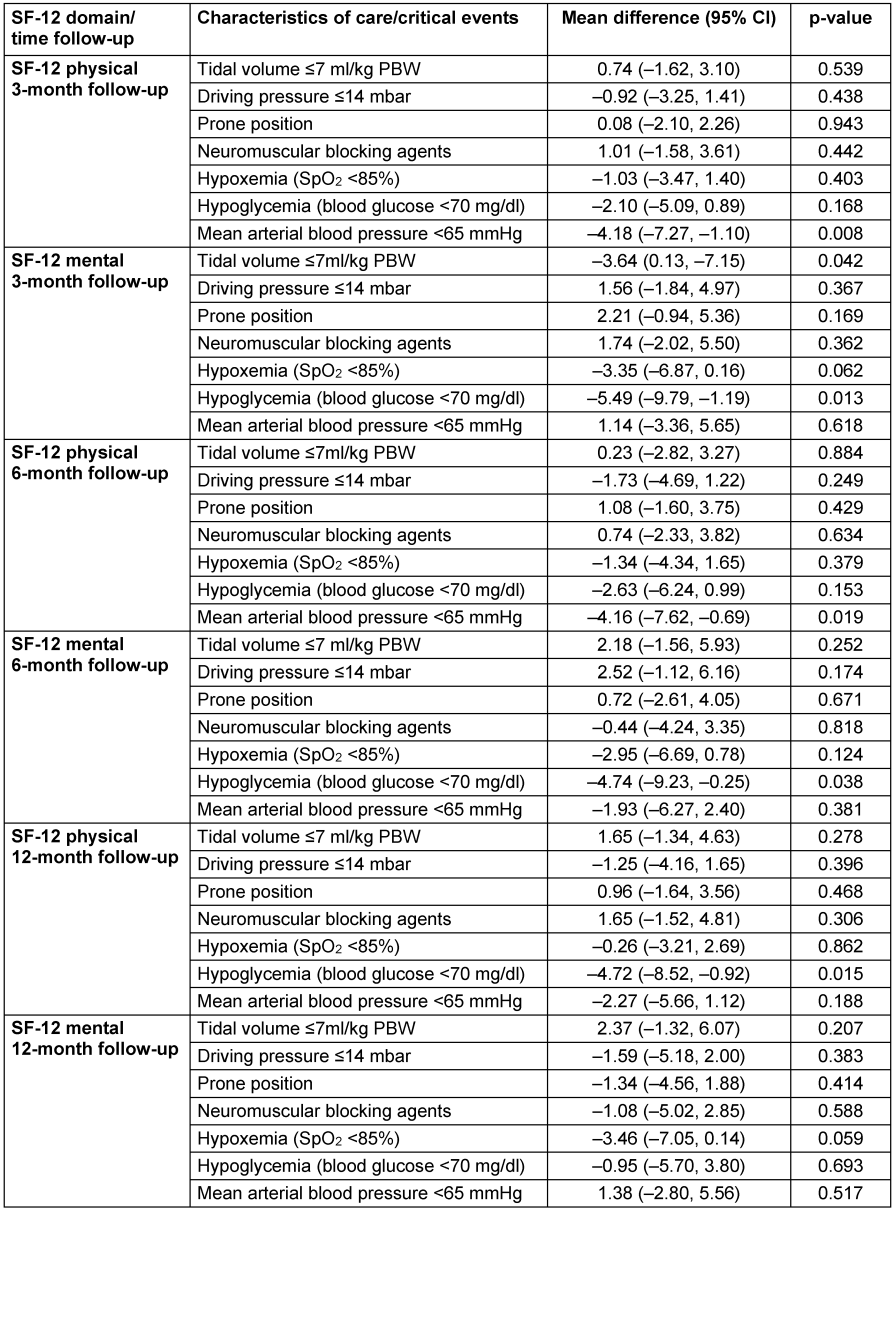
Association of acute care and critical events with 1-year health-related quality of life (SF-12); multiple analysis adjusted for SOFA (without Glasgow Coma Scale), body mass index, age and sex

**Table 4:**
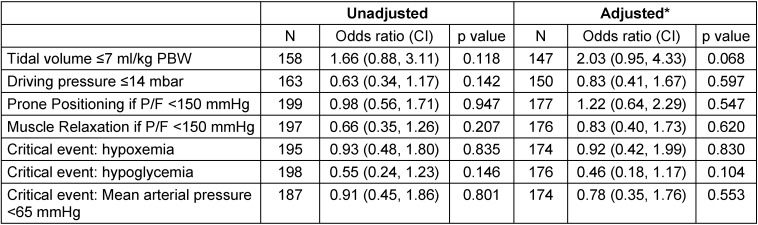
Effect of acute care management and critical events on return to work after 12 months: Multiple regression analysis (*adjusted for SOFA without Glasgow Coma Scale, body mass index, age and sex)
