# Supplementary material for: The quality of acute intensive care and the incidence of critical events have an impact on health-related quality of life in survivors of the acute respiratory distress syndrome – a nationwide prospective multicenter observational study
Source: Ger Med Sci. 2020 Jan 20;18:Doc01. doi: 10.3205/000277 (PMC6997802; doi:10.3205/000277)
Supplement: Acknowledgement [file GMS-18-01-s-001.pdf]

Acknowledgement

We are indebted to all the intensivists and study nurses throughout Germany, who, with great commitment, recruited patients for the DACAPO study:

Aachen, Aachen University Hospital RWTH Aachen, Department of Anesthesiology (PD Dr. Johannes Bickenbach, Dr. Thorben Beeker, PD Dr. Tobias Schürholz, Jessica Pezechk); Amberg, Klinikum Amberg, Department for Anaesthesiology (Dr. Jens Schloer); Augsburg, Klinikum Augsburg (Dr. Ulrich Jaschinski, Ilse Kummer); Bamberg, Sozialstiftung Bamberg Hospital, Department for Anaesthesiology (Dr. Oliver Kuckein); Berlin, Charité – University Medicine Berlin, Department of Anaesthesiology and Intensive Care Medicine (PD Dr. Steffen Weber-Carstens, Dr. Anton Goldmann, Dr. Stefan Angermair, Krista Stoycheva); Berlin, HELIOS Klinikum Berlin-Buch, Department of Intensive Care Medicine (Prof. Dr. Jörg Brederlau, Nadja Rieckehr, Gabriele Schreiber, Henriette Haennicke); Bielefeld, Ev. Krankenhaus Bielefeld. Department of Anesthesiology, Intensive Care Medicine, Emergency Medicine and Pain Therapy (Dr. Friedhelm Bach, Dr. Immo Gummelt, Dr. Silke Haas, Catharina Middeke, Dr. Ina Vedder, Marion Klaproth); Bochum, Ruhr University Bochum, Department of Anaesthesiology (Prof. Dr. Michael Adamzik, Dr. Jan Karlik, Dr. Stefan Martini, Luisa Robitzky); Bonn, University Hospital Bonn, Department of Anesthesiology and Intensive Care Medicine (Prof. Dr. Christian Putensen, Dr. Thomas Muders, Ute Lohmer); Bremen, Klinikum Bremen-Mitte, Department of Anesthesiology (Prof. Dr. Rolf Dembinski); Deggendorf, Medical Center, Department of Anaesthesiology and Intensive Care Medicine (Dr. Petra Schäffner, Dr. Petra Wulff-Werner); Dortmund, Klinikum Dortmund, Department of Critical Care Medicine (Elke Landsiedel-Mechenbier, Daniela Nickoleit-Bitzenberger, Ann-Kathrin Silber); Dresden, University Hospital Dresden Carl Gustav Carus, Department of Anesthesiology and Intensive Care Medicine (Prof. Dr. Maximilian Ragaller, Prof. Dr. Marcello Gama de Abreu, Alin Ulbricht, Linda Reisbach); Frankfurt am Main, University Hospital Frankfurt, Department of Anaethesiology, Intensive Care Medicine and Pain Therapy (Prof. Dr. Kai Zacharowski, Prof. Dr. Patrick Meybohm, Simone Lindau, Haitham Mutlak); Freiburg, University Medical Center Freiburg, Department of Anaesthesiology and Critical Care Medicine Freiburg (Prof. Dr. Alexander Hötzel, Dr. Johannes Kalbhenn); Freising, Klinikum Freising, Department of Anaesthesiology (Dr. Christoph Metz, Dr. Stefan Haschka); Göppingen, Klinik am Eichert, ALB FILS Kliniken, Department of Anaesthesiology and Intensive Care (Dr. Stefan Rauch); Göttingen, University Medical Center, Department of Anaesthesiology, Emergency and Intensive Care Medicine (Prof. Dr. Michael

Quintel, Dr. Lars-Olav Harnisch, Dr. Sophie Baumann, Andrea Kernchen); Greifswald, University Medicine Greifswald, Department of Internal Medicine B (Dr. Sigrun Friesecke, Sebastian Maletzki); Hamburg, University Hospital Hamburg-Eppendorf, Department of Intensive Care Medicine, Center of Anesthesiology and Intensive Care Medicine (Prof. Dr. Stefan Kluge, Dr. Olaf Boenisch, Dr. Daniel Frings, Birgit Füllekrug, Dr. Nils Jahn, Dr. Knut Kampe, Grit Ringeis, Brigitte Singer, Dr. Robin Wüstenberg); Hannover, Hannover Medical School, Department of Anaesthesiology and Intensive Care Medicine (Dr. Jörg Ahrens, Dr. Heiner Ruschulte, Dr. Andre Gerdes, Dr. Matthias Groß); Hannover, Hannover Medical School, Department of Respiratory Medicine (Dr. Olaf Wiesner, Aleksandra Bayat-Graw); Heidelberg, University of Heidelberg, Department of Anaesthesiology (Dr. Thorsten Brenner, Dr. Felix Schmitt, Anna Lipinski); Herford, Klinikum Herford, Clinic for Anaesthesiology, Surgical Intensive Care Medicine, Emergency Care Medicine, Pain Management (Prof. Dr. Dietrich Henzler, Dr. Klaas Eickmeyer, Dr. Juliane Krebs, Iris Rodenberg); Homburg, Homburg University Medical Centre, Department of Anaesthesiology, Intensive Care and Pain Medicine (Dr. Heinrich Groesdonk, Kathrin Meiers, Karen Salm, Prof. Dr. Thomas Volk); Ibbenbüren, Ibbenbüren General Hospital, Division of Thoracic Surgery and Lung Support (Prof. Dr. Stefan Fischer, Basam Redwan); Immenstadt, Kempten-Oberallgaeu Hospitals, Clinic for Pneumology, Thoracic Oncology, Sleep- and Respiratory Critical Care (Dr. Martin Schmölz, Dr. Kathrin Schumann-Stoiber, Simone Eberl); Ingolstadt, Klinikum Ingolstadt, Department of Anaesthesiology and Critical Care Medicine (Prof. Dr. Gunther Lenz, Thomas von Wernitz-Keibel, Monika Zackel); Jena, Jena University Hospital, Department of Anesthesiology and Intensive Care Therapy (Dr. Frank Bloos, Dr. Petra Bloos, Anke Braune, Anja Haucke, Almut Noack, Steffi Kolanos, Heike Kuhnsch, Karina Knuhr-Kohlberg); Kassel, Klinikum Kassel, Department of Anaesthesiology (PD Dr. Markus Gehling); Kempten, Klinikum Kempten-Oberallgäu gGmbH, Department for Anesthesia and Operative Intensive Care (Prof. Dr. Mathias Haller, Dr. Anne Sturm, Dr. Jannik Rossenbach); Kiel, University Medical Center Schleswig-Holstein, Campus Kiel, Department of Anesthesiology and Intensive Care Medicine (Dr. Dirk Schädler, Stefanie D'Aria); Köln, Cologne-Merheim Hospital, Department of Pneumology and Critical Care Medicine (Prof. Dr. Christian Karagiannidis, Dr. Stephan Straßmann, Prof. Dr. Wolfram Windisch); Köln, University Hospital of Cologne, Department of Anaesthesiology and Intensive Care Medicine (Prof. Dr. Thorsten Annecke, PD Dr. Holger Herff); Langen, Asklepios Kliniken Langen-Seligenstadt, Department of Anesthesiology and Intensive Care Medicine (Dr. Michael Schütz); Leipzig, University of Leipzig, Department of Anesthesiology and Intensive Care Medicine (PD Dr. Sven Bercker, Hannah Reising, Mandy Dathe, Christian Schlegel); Ludwigsburg, Klinikum Ludwigsburg, Academic

Teaching Hospital, University of Heidelberg, Department of Anaesthesiology (Katrin Lichy); Ludwigshafen, Klinikum Ludwigshafen, Department of Anesthesiology and Intensive Care Medicine (Prof. Dr. Wolfgang Zink, Dr. Jana Kötteritzsch); Mainz, University Medical Center Mainz, Department of Anaesthesiology (Dr. Marc Bodenstein, Susanne Mauff, Peter Straub); Magdeburg, Magdeburg University Medical Centre, Department of Anaesthesiology and Intensive Care Medicine (Dr. Christof Strang, Florian Prätsch, Prof. Dr. Thomas Hachenberg); Mannheim, University Medical Center Mannheim, Department of Anaesthesiology and Surgical Intensive Care Medicine (Dr. Thomas Kirschning, Dr. Thomas Friedrich, Dr. Dennis Mangold); Marburg, University Hospital, Department of Anaesthesiology (Dr. Christian Arndt, Tilo Koch); Mönchengladbach, Kliniken Maria-Hilf GmbH, Department of Cardiology (Dr. Hendrik Haake, Katrin Offermanns); München, Bogenhausen Hospital, Department of Anaesthesiology (Prof. Dr. Patrick Friederich, Dr. Florian Bingold); München, Klinikum Großhadern, Department of Anaesthesiology (Dr. Michael Irlbeck, Prof. Dr. Bernhard Zwissler); München, Klinikum Neuperlach, Städtisches Klinikum München GmbH, Department of Anesthesiology, Critical Care and Pain Medicine (PD Dr. Ines Kaufmann); München, Klinikum rechts der Isar, Department for Anaesthesiology of the Technical University of Munich (Dr. Ralph Bogdanski, Dr. Barbara Kapfer, Dr. Markus Heim, PD Dr. Günther Edenharter); Münster, University Hospital Münster, Department for Anaesthesiology, Intensive Care Medicine and Pain Therapy, (Prof. Dr. Björn Ellger, Daniela Bause); Neumarkt, Kliniken des Landkreises Neumarkt i.d.OPf, Department for Anaesthesiology and Intensive Care Medicine (Dr. Götz Gerresheim); Nürnberg, General Hospital Nuremberg, Paracelsus Medical University, Department of Emergency Medicine and Intensive Care (Dr. Dorothea Muschner, Prof. Dr. Michael Christ, Arnim Geise); Osnabrück, Marienhospital Osnabrück, Department of Anaesthesiology (PD Dr. Martin Beiderlinden, Dr. Thorsten Heuter); Passau, Klinikum Passau, Department for Anaesthesiology (Dr. Alexander Wipfel); Regensburg, Caritas Krankenhaus St. Josef, Department for Anaesthesiology (Dr. Werner Kargl, Dr. Marion Harth, Dr. Christian Englmeier); Regensburg, Regensburg University Hospital, Department of Anaesthesiology, Operative Intensive Care (Prof. Dr. Thomas Bein, Dr. Sebastian Blecha, Dr. Kathrin Thomann-Hackner, Marius Zeder); Stuttgart, Katharinenhospital, Department of Anesthesiology (Dr. Markus Stephan); Traunstein, Klinikum Traunstein, Department of Anaesthesiology (Dr. Martin Glaser); Tübingen, Tübingen University Hospital, Eberhard-Karls University Tübingen, Department of Anaesthesiology and Intensive Care Medicine (PD Dr. Helene Häberle); Ulm, Ulm University, Department of Anesthesiology (Prof. Dr. Hendrik Bracht, Christian Heer, Theresa Mast); Würzburg, University of Würzburg, Department of Anaesthesia and Critical Care (PD Dr. Markus Kredel, PD Dr. Ralf Müllenbach)

Further, we are grateful to previous members of the Regensburg DACAPO study team (medical documentation: Phillip Sebök, study physician: Kathrin Thomann-Hackner), to the members of the Advisory Board of the DACAPO-Study (Prof. Dr. Julika Loss, Prof. Dr. Bernhard Graf, Prof. Dr. Michael Leitzmann, Prof. Dr. Michael Pfeifer, Regensburg, Germany) and to our student assistants (Simon Bein, Vreni Brunnthaler, Carina Forster, Stefanie Hertling, Sophie Höhne, Carolin Schimmele, Elisa Valetta). We are grateful to Prof. Arthur Slutsky, Toronto, for critically reviewing the intellectual content of an earlier version of this manuscript.
